# Multi-omics reveal the metabolic patterns in mouse cumulus cells during oocyte maturation

**DOI:** 10.1186/s13048-023-01237-8

**Published:** 2023-08-08

**Authors:** Ming Chen, Weizheng Yang, Yueshuai Guo, Xiaojing Hou, Shuai Zhu, Hongzheng Sun, Xuejiang Guo, Minjian Chen, Qiang Wang

**Affiliations:** 1 State Key Laboratory of Reproductive Medicine, Suzhou Municipal Hospital, Nanjing Medical University, 101 Longmian Rd, Nanjing, Jiangsu, 211166 China; 2 Women’s Hospital of Nanjing Medical University, Nanjing Maternity and Child Health Care Hospital, Nanjing, China; 3 Key Laboratory of Modern Toxicology of Ministry of Education, School of Public Health, Nanjing Medical University, Nanjing, China; 4 Department of Histology and Embryology, Nanjing Medical University, Nanjing, 211166 China; 5 Center for Global Health, School of Public Health, Nanjing Medical University, Nanjing, 211166 China

**Keywords:** Cumulus cells, Oocyte, Metabolomics, Proteomics, Reproduction

## Abstract

Bi-directional communication between cumulus cells and the surrounded oocytes is important for the development and functions of both compartments. However, the metabolic framework in cumulus cells has not been systematically described. In the present study, cumulus cells from cumulus-oocyte complexes (COCs) at three key time points were isolated (arrested GV stage, post-hCG 0h; meiotic resumption GVBD stage, post-hCG 3h; and metaphase II stage, post-hCG 12h), and the temporal metabolomic and proteomic profiling were performed. Integrated multi-omics analysis reveals the global metabolic patterns in cumulus cells during mouse oocyte maturation. In particular, we found the active hyaluronic acid metabolism, steroid hormone synthesis, and prostaglandin E2 (PGE2) production in cumulus cells. Meanwhile, accompanying the oocyte maturation, a progressive increase in nucleotide and amino acid metabolism was detected in the surrounding cumulus cells. In sum, the data serve as a valuable resource for probing metabolism during terminal differentiation of ovarian granulosa cells, and provide the potential biomarkers for improving and predicting oocyte quality.

**Supplementary Information:**

The online version contains supplementary material available at 10.1186/s13048-023-01237-8.

## Introduction

A crucial objective of folliculogenesis is the production of a fertilized egg capable of undergoing activation and embryogenesis. In order to achieve this goal, there is precise coordination between oocytes and cumulus cells. After puberty, groups of oocytes are recruited to the growth phase to produce developmentally competent, transcriptionally silent, fully grown germinal vesicle (GV) [1]. Following the induction by luteinizing hormone, GV oocytes acquire the ability to resume meiosis I. It is characterized by germinal vesicle breakdown (GVBD) [2]. Meiosis I begins with the assembly of the meiotic spindle and ends when the oocyte releases its first polar body [3]. Early-arrested germinal vesicle oocytes resume meiosis and enter the second meiotic metaphase (referred to as MII oocytes), completing a process commonly referred to as oocyte maturation [3, 4]. Under physiological conditions, oocytes are typically enclosed in a fluid microenvironment of several layers of cumulus cells and follicular fluid.​ During the pre-ovulatory period, the cumulus cell changes from a compact cell mass to a decentralized cell structure, allowing for the synthesis and deposition of a mucoid intercellular matrix, a process referred to as cumulus expansion [5]. The proximity of these cells to each other allows bidirectional communication between oocytes and the surrounding somatic cells, and thus the meiotic maturation of oocytes is typically accompanied by the correct execution of cumulus cell functions [6]. Metabolites and metabolism-related enzymes are participants of metabolism that provide a snapshot of the wellbeing of cumulus-oocyte complexes and the mechanisms that control key processes of oocyte maturation. Thus, exploring the metabolism in cumulus cells is key to understand the modulation of oocyte development.

The lower success rate of current in vitro maturation (IVM) systems compared to in vivo-matured oocytes is in part attributed to its suboptimal culture conditions that do not adequately support the COCs’ metabolic needs [7]. Metabolic disorders including diabetes, obesity and polycystic ovary syndrome have been reported to cause meiotic abnormality in oocytes and ovulation dysfunction, ultimately leading to subfertility and even infertility [8, 9]. The metabolic cooperativity between cumulus cells and oocytes has been implicated to be involved in these events [10–14]. ​Although some metabolites and metabolism-related enzymes have been reported, a dynamic multi-omics-based metabolic profile of mouse cumulus cells could better help us understand the development of cumulus cells.

In the present study, we obtained the dynamic metabolic profile of mouse cumulus cells during in vivo maturation. In parallel, quantitative proteomic analysis identified the key enzymes responsible for the metabolism in cumulus cells. The construction of the metabolic network may provide an opportunity to better understand the developmental control of oocytes.

## Materials and methods

### Mouse

All experiments were approved by the local animal ethical committee and the Animal Care and Use Committee of Nanjing Medical University (Protocol NO. IACUC-1703017). Female C57BL/6 mice were purchased from the experimental animal center of Nanjing Medical University, and were housed in ventilated cages on a 12–12/h light/dark cycle at constant temperature (22 °C) and under controlled humidity (under SPF specified pathogen free conditions). Five mice were randomly selected from each cage, and all cages were housed under the same conditions (including appropriate density, clean bedding and cleaning frequency). The mice were euthanized with carbon dioxide, followed by cervical dislocation before ovary collection.

### Cumulus cells collection

Three-week-old female mice were first injected with 5 units of pregnant horse serum gonadotropin (PMSG), followed by 5 units of human chorionic gonadotropin (hCG) 48 h after the onset of PMSG superovulation. Mice were sacrificed by cervical dislocation at 0, 3 and 12 h after hCG injection. To collect cumulus cells surrounding GV and GVBD oocytes, cumulus-oocyte complexes were retrieved by manual rupturing of antral ovarian follicles, and cumulus cells were separated by repeatedly pipetting. To collect cumulus cells surrounding MII oocytes, COCs were isolated from oviduct ampullae, and cumulus masses were separated in M16 (Sigma-Aldrich, Cat# M7292) medium containing 0.5 mg/ml^−1^ hyaluronidase at 37 °C. ​

### Metabolomics

Metabolome data were collected and analyzed as described previously [15]. Cumulus cells were harvested separately from 2, 000 GV, GVBD and MII COCs for each sample (~ 2.4 × 10^6^ cumulus cells per sample, 9 samples for each stage). Samples were transferred to Eppendorf tubes, immediately flash frozen in liquid nitrogen, and then stored at -80 °C. For metabolite extraction, samples were resuspended in 300 mL of 80% methanol/water (vol/vol) and homogenized using an Ultra-Turrax homogenizer. After cooling on ice for 10 min, samples were spun at 16,000 g for 15 min at 4 °C. Supernatant (250 mL per sample) was transferred to a new tube, and dried samples were stored at -80 °C. until instrumental analysis. The metabolomics data acquisition was conducted using a standard metabolic profiling method we previously described. Briefly, experiments were conducted on an UPLC Ultimate 3000 system (Dionex, Germering, Germany) coupled to a Q-Exactive mass spectrometer (Thermo Fisher Scientific, Bremen, Germany). All samples were analyzed in a randomized fashion to avoid complications caused by injection order. The chromatographic separation was performed with Hypersil GOLD C18 column (100 mm × 2.1 mm, 1.9 μm) (Thermo Fisher Scientific) at 40 °C. with at a flow rate of 0.4 mL/min. The mobile phase consisted of phase A (0.1% formic acid in pure ACN) and phase B (0.1% formic acid in ultra-purewater). After the initial 3-min elution of 1% (vol/vol) A, the percentage of solvent A gradually increased to 99% (vol/vol) at t = 10 min. The solvent A was maintained at 99% (vol/vol) for 3 min (t = 13 min), and then immediately decreased to 1% (vol/vol) lasting for 2 min (t = 15 min). The mass spectrometer analysis was conducted at a 70,000 resolution in both positive and negative modes, and a full-scan mode ranging from 70 m/z to 1,050 m/z was used.

Raw data obtained from the mass spectrometer were submitted to TraceFinder v3.1 (Thermo Fisher Scientific). The metabolite identification was conducted by the comparison of accurate mass and retention time with the commercial standard compounds using the author-constructed library. All statistical analyses were performed with ‘‘R’’ (V4.2.0). Student’s t test was used for comparing continuous variables between two groups [16]. OPLS-DA was conducted by SIMCA-P software (V14.0; Umetrics AB, Umea, Sweden). The combined cut-offs of the statistical significance including the variable importance in projection (VIP) value > 1.00 and *P* value < 0.05 of each metabolite were used. KEGG Mapper (V4.1) was used for the integration and visualization of metabolomics and proteomics data. The oocyte metabolomics data used in this paper are all from our previously published study [15].

### Proteomics

Proteome data were collected and analyzed as described previously [15, 17]. GV, GVBD, and MII cumulus cells collected from normal mice were lysed in urea lysis buffer (8 M urea, 75 mM NaCl, 50 mM Tris, pH 8.2, 1%(v/v) EDTA-free protease inhibitor, 1 mM NaF, 1 mM b-glycerophosphate, 1 mM sodium orthovanadate, 10 mM sodium pyrophosphate, 1 mM Phenylmethylsulfonyl fluoride (PMSF)) and subjected to centrifugation at 40,000 g for 1 h. Protein content was measured using the Bradford method. Proteins were reduced, alkylated and trypsin digested as we described before [15]. Following digestion, the purified peptides were subjected to a 9-plex experiment using the TMT10plex™ kit according to the manufacturer's protocols. Peptides from three replicates of three stages of cumulus cells (cumulus cells surrounding 375 oocytes for one replicate of each stage of cumulus cells) were labeled with respective isobaric tags and mixed together. To improve the coverage of protein identification and quantification, the mixed TMT-labeled peptides were separated by the high-pH reversed phase (HP-RP) fractionation technology based on the ACQUITY®UPLC M-class system (Waters) with an BEH C18 Column (300 μm × 150 mm, 1.7 μm; Waters), and a total of 25 fractions were collected and lyophilized. All fractions were sequentially reconstituted in 0.1% FA analyzed using an Orbitrap Fusion Lumos mass spectrometer (ThermoFisher Scientific) coupled to a Proxeon Easy-nLC 1200 system. Peptides were separated with an analytical column (75 μm × 160 mm, 1.9 μm, Dr. Maisch) using a 95 min linear gradient (3% to 5% buffer B for 5 s, 5% to 15% buffer B for 40 min, 15% to 28% buffer B for 34 min and 50 s, 28% to 38% buffer B for 12 min, 38% to 100% buffer B for 5 s, 100% buffer B for 8 min) at 300 nl/min. The parameter settings for MS could be found in previously published paper [17].

The MaxQuant software (V1.6.5.0) was used for searching the raw files against UniProt mouse proteome database [18]. The FDR (false discovery rate) of identified peptides and proteins was set to 1%. Precursor mass tolerance was set to 20 ppm and product ions were searched with a mass tolerance 0.5 Da. Searches were performed using Trypsin/P enzyme specificity while allowing up to two missed cleavages. Carbamidomethylation of cysteine residues (+ 57.0215 Da) were set as fixed modifications. Variable modifications included oxidation of methionines and acetylation of protein N termini. For TMT settings, the protein quantification values were calculated using the reporter ion MS2 method of isobaric labels in MaxQuant. Peptides and proteins with FDR ≤ 1% were considered, and proteins with at least one unique peptide were subjected to quantitative analysis.

For GV, GVBD, and MII cumulus cells, Student’s t test was performed, and a protein with *p* < 0.05 and fold change > 1.5 was considered significant differences in abundances between groups. Heatmap was produced accompanied by a dendrogram depicting the extent of similarity of protein expression among the samples (The heatmap was created in RStudio (4.2.0), package: ComplexHeatmap) [19]. For the convenience of gene annotation, corresponding Ensembl gene IDs of the differentially expressed proteins were used for further bioinformatics analysis. Principal component analysis was conducted using finalized Principal component analysis was conducted using finalized differential protein (R package: FactoMineR) [20]. To characterize these genes, enrichment of Kyoto Encyclopedia of Genes and Genomes (KEGG) pathways was analyzed by using the clusterProfiler R package [21]. Flowchart of metabolomics and proteomics adapted from Servier Medical Art (https://smart.servier.com/).

### Statistical analysis

Each experiment was repeated at least three times, and unless specified, the data shown in figure are the results of one representative experiment. The profiling of statistics was performed by the software GraphPad Prism (V 8.0.2) for Windows. Data are presented as means ± SD, unless otherwise stated. A probability value of < 0.05 was considered to denote statistical significance.

## Results

### Metabolomic and proteomic profiling of mouse cumulus cells

**Fig. 1:**
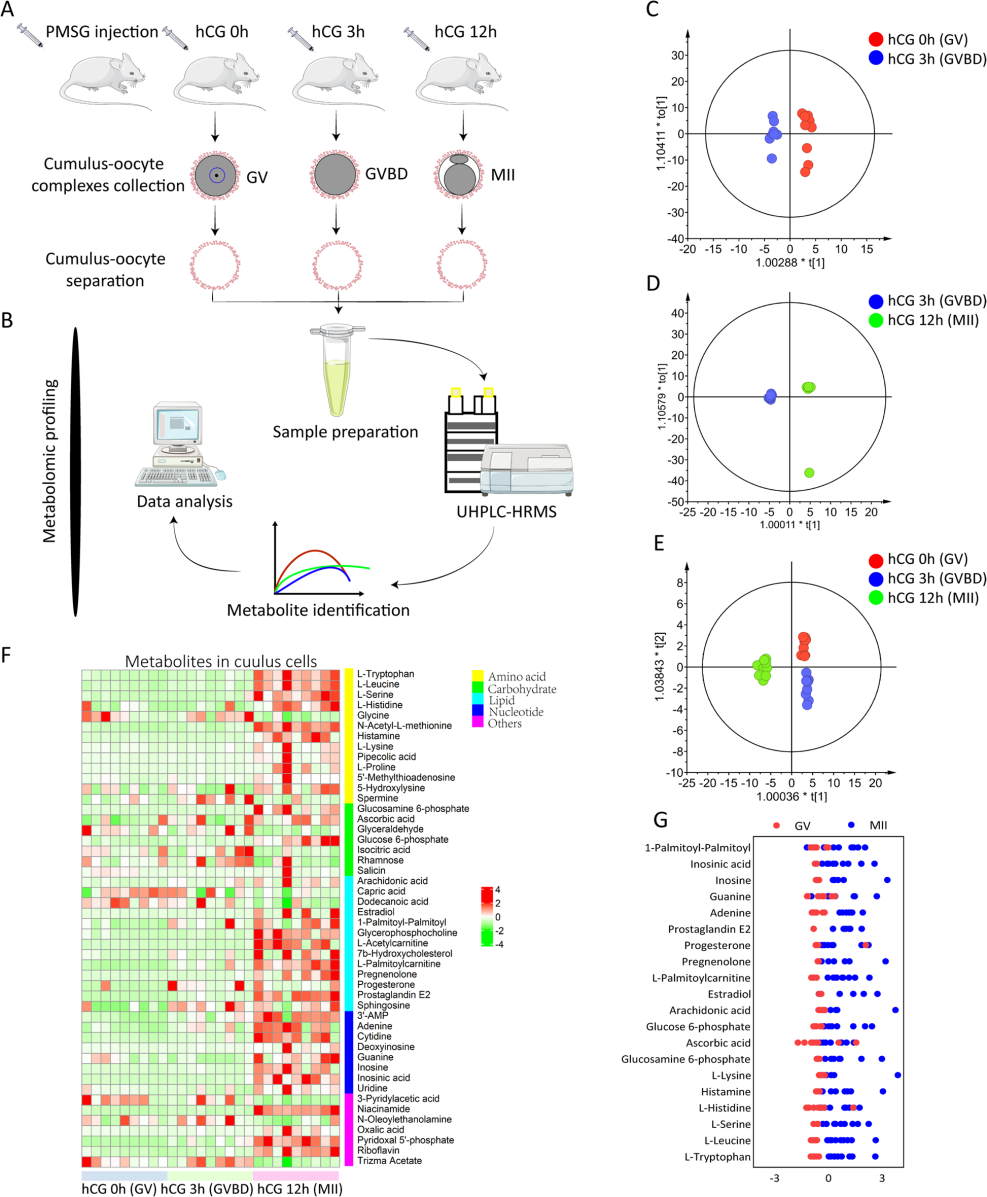
Metabolomic profiling of mouse cumulus cells. **A** Illustration of in vivo isolation of mouse cumulus cells at three key time points. **B** Workflow for UPLC/MS-based metabolome profiling on mouse cumulus cells. **C**-**E** OPLS-DA score plot for metabolomic datasets clearly distinguishes cumulus cell samples from three time points. **F** Heatmap visualizing the relative abundance of differential metabolites in cumulus cells during oocyte maturation. **G** Z-score plot of 20 representative metabolites from **F** compared between GV and MII cumulus cells. Each dot represents one test and categorized by different colors. The complete metabolomic data are available in Supplementary Table 1

Cumulus cells surrounding the oocytes are tightly packed in layers forming the cumulus-oocyte complex (COCs). They serve to provide nutrition to the oocytes and operate as a network between the oocyte and its extracellular microenvironment [22]. Here, we collected the large numbers of mouse cumulus cells at three key time points during oocyte maturation (post-hCG 0h, 3h and 12h correspond to GV, GVBD and MII, respectively), and analyzed the intracellular metabolome using ultra-high-performance liquid chromatography-tandem high-resolution mass spectrometry (UHPLC-HRMS) (Fig. 1A and B). We detected 48 differential metabolites in all, based on a t test (*p* < 0.05) coupled with a variable importance in projection (VIP) analysis (VIP > 1.00). Robust orthogonal partial least-squares-discriminant analysis (OPLS-DA) clearly showed the stage-dependent separation of three cumulus cells groups with the cross validated predictive ability of Q2(cum) ≥ 0.686 (Fig. 1C–E). The differential metabolites were mapped to their respective biochemical pathways, delineated in Kyoto Encyclopedia of Genes and Genomes (KEGG), revealing the distinct changing patterns (Fig. 1F) (Supplementary Table 1). Most of the differential metabolites increased in cumulus cells along with the oocyte maturation. 20 representative metabolites are shown in Fig. 1G.

**Fig. 2:**
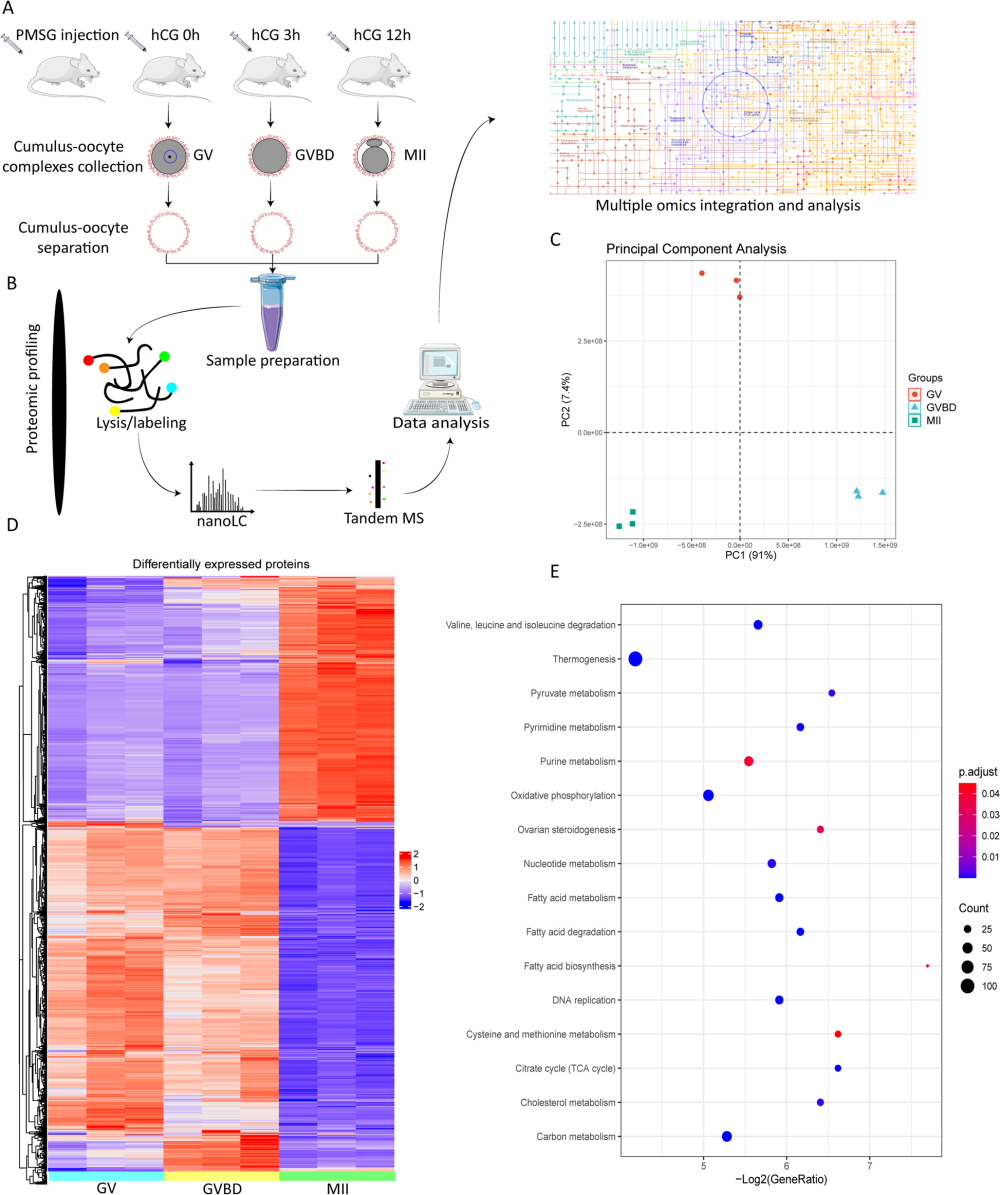
Proteomic profiling of mouse cumulus cells. **A**-**B** Schematic overview of the workflow for proteome profiling in cumulus cells. **C** Principal component analysis of cumulus cells heterogeneity. **D** Heatmap of 1,407 differentially expressed proteins among GV, GVBD, MII cumulus cells. **E** Bubble chart of enriched KEGG pathway terms for the differentially expressed proteins in cumulus cells during maturation. The complete proteomic data are available in Supplementary Table 1

Changes in a single metabolite cannot completely reflect the metabolic activity in cumulus cells. Metabolic enzymes are the mediators of cellular metabolism. To determine whether changes in substrates/products are correlated with a corresponding alteration in enzymatic protein accumulation in cumulus cells, we simultaneously conducted a quantitative proteomic analysis of GV, GVBD, and MII cumulus cells (Fig. 2A-B). Nine thousand three hundred thirty-six total proteins were identified, and 1,407 proteins with differential levels were discovered (false discovery rate [FDR] = 0.05). To evaluate the samples at different time points, principal component analysis was used to test the model effectiveness (Fig. 2C) (Supplementary Table 2). KEGG analysis showed a significant enrichment of categories related to metabolic pathways, such as purine metabolism and ovarian steroidogenesis (Fig. 2D-E) (Figure S1). In the following section, the metabolomic and proteomic data were integrated to characterize the metabolic features in cumulus cells during oocyte maturation.

### Carbohydrate metabolism in cumulus cells

**Fig. 3:**
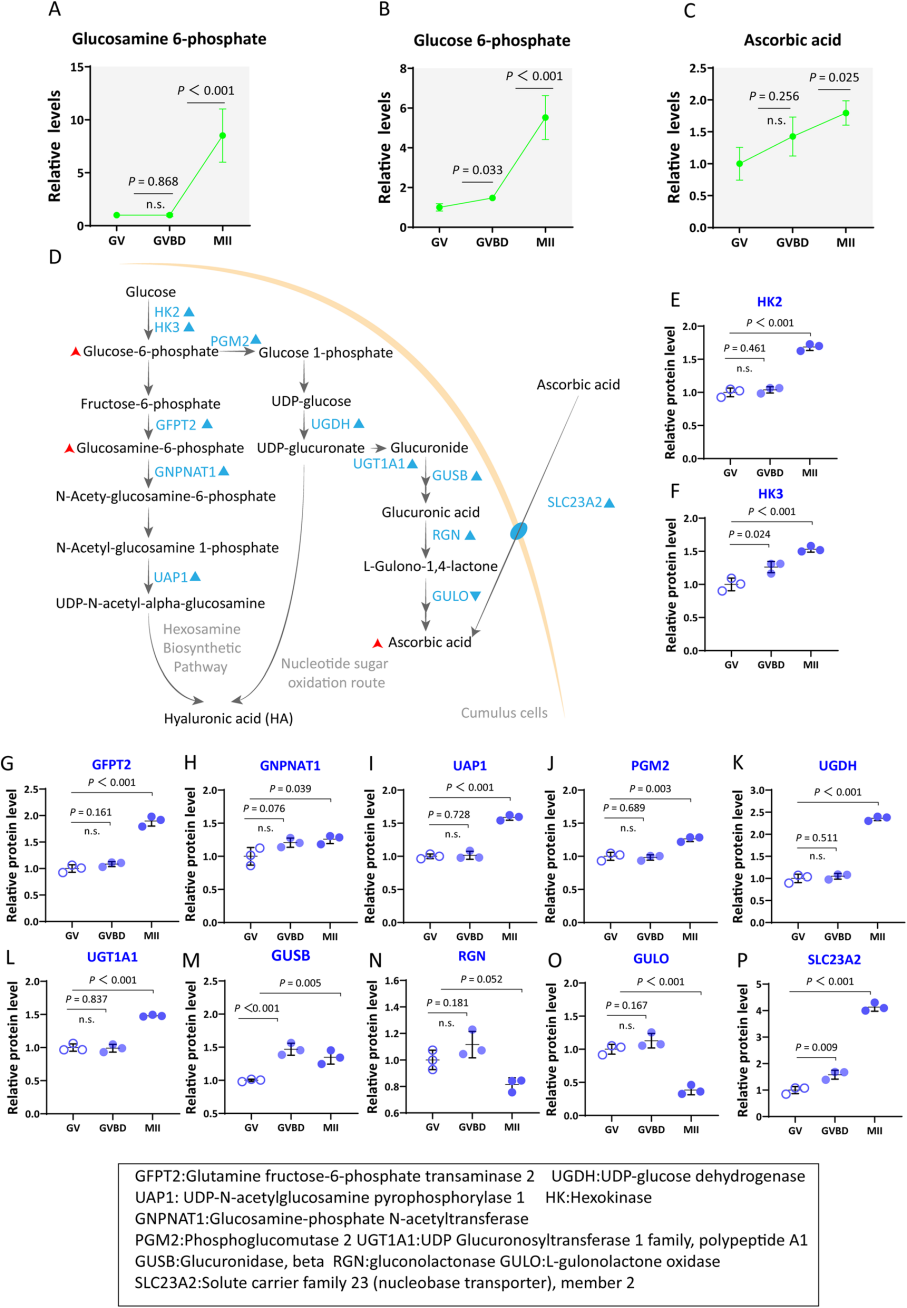
Active hyaluronic acid metabolism and elevated ascorbic acid in cumulus cells during oocyte maturation. **A**-**C** Relative levels of metabolites in cumulus cells at three time points. **D** Schematic diagram of hyaluronic acid and ascorbic acid metabolism. Increased metabolites in cumulus cells during meiotic resumption are indicated by bold red arrows. Differential metabolic enzymes changes are indicated by blue triangles. **E**-**P** Relative abundance of the representative enzymes involved in hyaluronic acid and ascorbic acid metabolism. Error bars, SD. Student’s t test was used for statistical analysis in all panels, comparing to GV cumulus cells. n.s., not significant

It has been implicated that cumulus cells play a significant role in the carbohydrate metabolism during oocyte maturation. We found that the levels of glucose 6-phosphate, ascorbic acid and glucosamine 6-phosphate were increased in cumulus cells during maturation (Fig. 3A-C). In contrast, isocitric acid and rhamnose are elevated during meiotic resumption and then declined in MII cumulus cells. Such the dynamic shift of carbohydrate-related products might play a role in oocytes maturation, ovulation, and even sperm − oocyte binding [23].

#### Active hyaluronic acid metabolism in cumulus cells during oocyte maturation

During the process of cumulus expansion, cumulus cells secrete hyaluronidase-sensitive mucous material, which is composed of proteoglycans and glycosaminoglycans. The major structural macromolecule in the matrix of the expanded cumulus cells is hyaluronic acid, a glycosaminoglycan composed of repeated disaccharides of glucuronic acid and N-acetyl-glucosamine [24]. Hyaluronan accumulation within the COCs during cumulus expansion is a necessary step in the maturation process [25]. Hexokinase 2 (HK2) and Hexokinase 3 (HK3) converts glucose to glucose-6-phosphate, the first committed step in glucose metabolism. Glucose-6-phosphate forms multiple metabolites under different conditions and shunts into differential metabolic pathways (i.e., Glycolysis, Pentose phosphate pathway, Hexosamine biosynthetic pathway, Nucleotide sugar oxidation route) (Fig. 3D) [26]. Proteomic profiles showed the increased levels of enzymes related to the hexosamine biosynthetic pathway (GFPT2, GNPNAT1 and UAP1) and the nucleotide oxidation route (PGM2 and UGDH) (Fig. 3E-P). The hexosamine biosynthetic pathway entails the transfer of glucose 6-phosphate into glucosamine-6-phosphate, which could be utilized for UDP-N-acetyl-glucosamine synthesis. Meanwhile, nucleotide sugar oxidation route generates, as ultimate product, the UDP-glucuronate. UDP-N-acetyl-glucosamine and UDP-glucuronate were the substrates of hyaluronic acid. Collectively, these data indicate that there is active hyaluronic acid synthesis in cumulus cells during meiotic maturation.

#### Elevated ascorbic acid in cumulus cells during oocyte maturation

Ascorbic acid, commonly known as vitamin C, is an essential dietary nutrient that is necessary for a variety of physiological processes in human body, including wound repair and collagen production [27]. Most animals are able to synthesize their own vitamin C. However, apes (including humans), monkeys (but not all primates), and some rodents must acquire it from dietary sources [28]. Meanwhile, ascorbic acid has a beneficial effect on germ cell population maintenance in the organoids [29]. We found that the ascorbic acid level was increased in the surrounding cumulus cells during oocyte maturation (Fig. 3C). Two sources of ascorbic acid may exist in cumulus cells: synthesis from UDP-glucose and take up from follicular fluid. By analyzing the proteomic data, we noticed the expression two important proteins, including L-gulonolactone oxidase (GULO) and solute carrier family 23 (nucleobase transporter) member 2 (SLC23A2). GULO is a key enzyme in the ascorbic Acid biosynthesis, remarkably, it was significantly reduced in cumulus cells during oocyte maturation (Fig. 3O). In contrast, SLC23A2, a Na^+^-dependent ascorbic acid transporter, was dramatically elevated in cumulus cells (Fig. 3L). Hence, it is conceivable that the increased ascorbic acid in cumulus cells during oocyte maturation is likely due to the elevated SLC23A2 expression, rather than the synthesis pathway through GULO.

### Lipid metabolism in cumulus cells

**Fig. 4:**
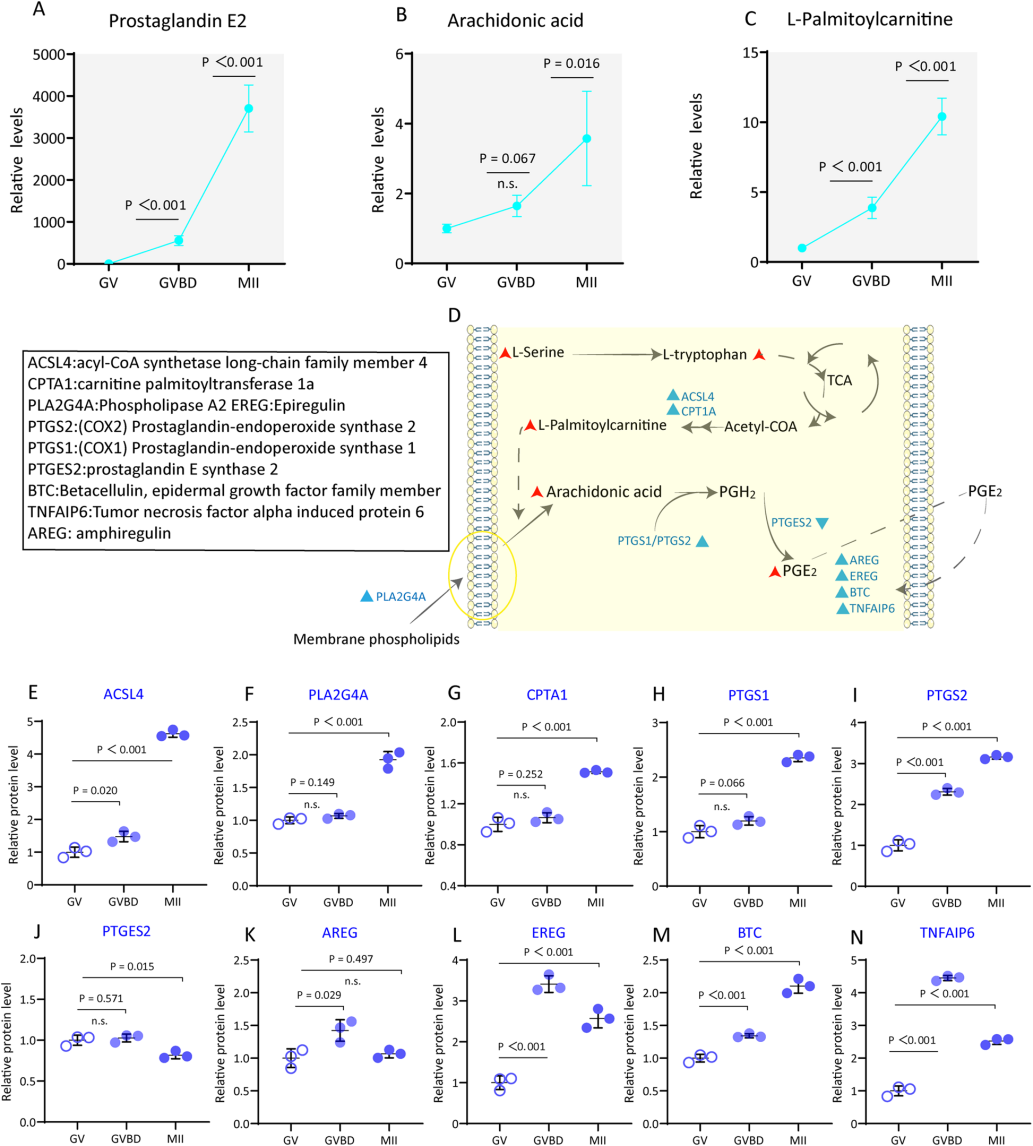
Increased PGE2 generation in cumulus cells during oocyte maturation. **A**-**C** Relative levels of metabolites related to PGE2 synthesis in cumulus cells. **D** Schematic diagram of PGE2 synthesis in cumulus cells during maturation. Increased metabolites in cumulus cells during meiotic resumption are indicated by bold red arrows. Differential metabolic enzymes changes are indicated by blue triangles. **E**-**N** Relative abundance of the representative enzymes involved in PGE2 synthesis. Error bars, SD. Student’s t test was used for statistical analysis in all panels, comparing to GV cumulus cells. n.s., not significant

It has been widely accepted that active lipid utilization in cumulus cells is essential for oocyte maturation [30]. ​However, further studies are needed to explore the dynamics of lipid metabolism in cumulus cells during oocyte maturation. Here, we found that the abundance of most lipid-related metabolites was increased in cumulus cells, such as prostaglandin E2 (PGE2), pregnenolone, L-palmitoylcarnitine and arachidonic acid (Fig. 4A-C).

#### Increased PGE2 generation in cumulus cells during oocyte maturation

Prostaglandin E2 (PGE2), an essential endogenous lipid mediator for normal physiological functions, also acts as an inflammatory mediator in pathological conditions [31]. In the past few decades, it has been known that PGE2 is one of the important determinants of ovulation. Nonetheless, the metabolic dynamics of PGE2 in the cumulus cells during oocyte maturation still needs further research [32, 33].

During COCs maturation, we found that PGE2 was dramatically elevated in cumulus cells (GVBD/GV: ~ 555-fold, MII/GV: ~ 3,705-fold), implying its significant effects on oocyte development (Fig. 4A). Prostaglandin H2 (PGH2) is derived from arachidonic acid by prostaglandin-endoperoxide synthase (PTGS1 or PTGS2), both enzymes showed increased expression (Fig. 4 H and I). PGH2 is further converted to PGE2 by one of three prostaglandin-E synthases [33], only PTGES2 showed changes in expression in the current study (Fig. 4J). Arachidonic acid can be cleaved from membrane phospholipids by the cytosolic phospholipase A2 (PLA2G4A). Meanwhile, L-Palmitoylcarnitine has been implicated to enhance the release of free arachidonic acid from intracellular membranes (Fig. 4D) [34]. Of note, proteomic profiles that an array of enzymes related revealed to PGE2 production was significantly accumulated in cumulus cells during meiotic resumption (Fig. 4E-J). Our proteomic data also showed that the downstream factors associated with PGE2 (i.e., epiregulin (EREG), betacellulin, epidermal growth factor family member (BTC), tumor necrosis factor alpha induced protein 6 (TNFAIP6) and amphiregulin (AREG)) were also increased in cumulus cells during oocyte maturation (Fig. 4K-N) [35]. Multiple events including ovulation have been reported to resemble inflammatory response. Unlike pathological inflammations, such “physiological” inflammations must be well controlled to avoid pathological consequences such as autoimmunity [36]. PGE2 is a well-known inflammatory marker, and its overproduction plays an important role in the inflammatory process [37]. Together, these data clearly show that PGE2 is synthesized in cumulus cells, and which might be essential for its own function by autocrine secretion, and beneficial for oocyte development and ovulation through paracrine secretion.

#### Steroid hormones are upregulated in cumulus cells

**Fig. 5:**
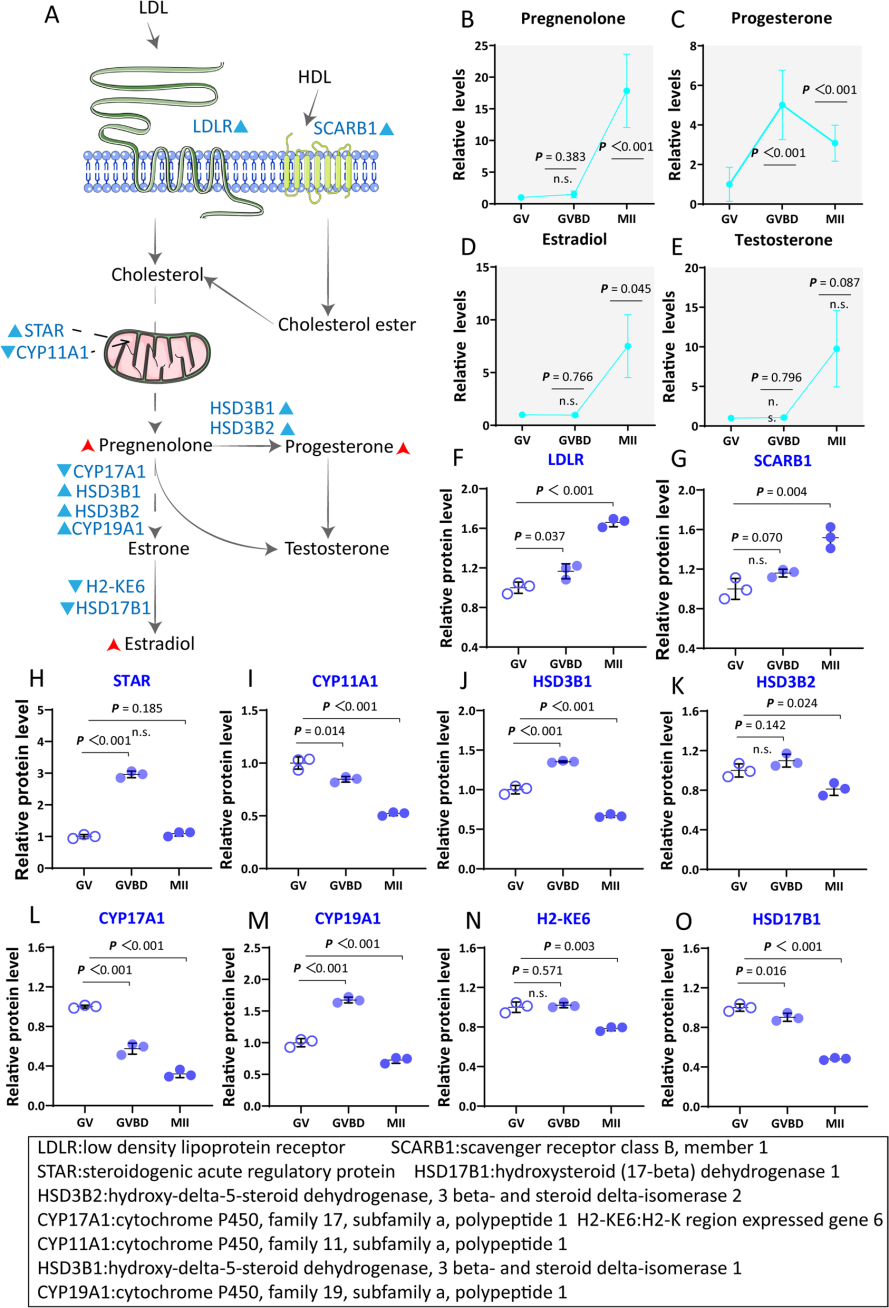
Active biosynthesis of steroid hormones in cumulus cells. **A** Schematic diagram of the biosynthesis of steroid hormones in the cumulus cells during maturation. Increased metabolites in cumulus cells during meiotic resumption are indicated by bold red arrows. Differential metabolic enzymes changes are indicated by blue triangles. **B**-**E** Relative levels of metabolites related to steroid hormone biosynthesis in cumulus cells at three time points. **F**-**O** Relative abundance of the representative enzymes involved in biosynthesis of steroid hormones. Error bars, SD. Student’s t test was used for statistical analysis in all panels, comparing to GV cumulus cells. n.s., not significant

Progesterone (P4) is an endogenous steroid and progestogen sex hormone involved in the menstrual cycle, pregnancy, and embryogenesis [38]. However, the role of P4 in regulating meiotic resumption is still controversial [39]. Our metabolomic analysis showed that P4 was enhanced from GV to GVBD stage and then declined from GVBD to MII stage in cumulus cells (Fig. 5C). In parallel, proteomic data showed that two enzymes involved in P4 synthesis (hydroxy-delta-5-steroid dehydrogenase, 3 beta- and steroid delta-isomerase 1/2 (HSD3B1/2)) exhibited the similar accumulation trend (Fig. 5J-K). Therefore, we believe that P4 synthesis in cumulus cells participates in the control of oocyte maturation.

Previous research has suggested that estradiol (E2) and testosterone (T) can promote the maturation of oocytes [40, 41]. Here, we found that the abundance of E2 and testosterone underwent a remarkable increase in cumulus cells surrounding MII oocytes (E2: ~ 7.5-fold; testosterone: ~ 9.7-fold) (Fig. 5D-E). Serum estradiol peaking just before ovulation which is the parallels the remarkable increase in E2 and T in cumulus cells post-hCG 12h [41–44]. Here, our proteomic profiles identified the increased accumulation of 6 (i.e., LDLR, SCARB1, STAR, HSD3B1, HSD3B2 and CYP19A1) out of 10 enzymes related to the metabolic pathways mentioned above (Fig. 5F-O). Cumulatively, our results suggest that steroid hormones in cumulus cells may promote oocyte development.

### Progressive increase in nucleotide metabolism in cumulus cells during oocyte maturation

**Fig. 6:**
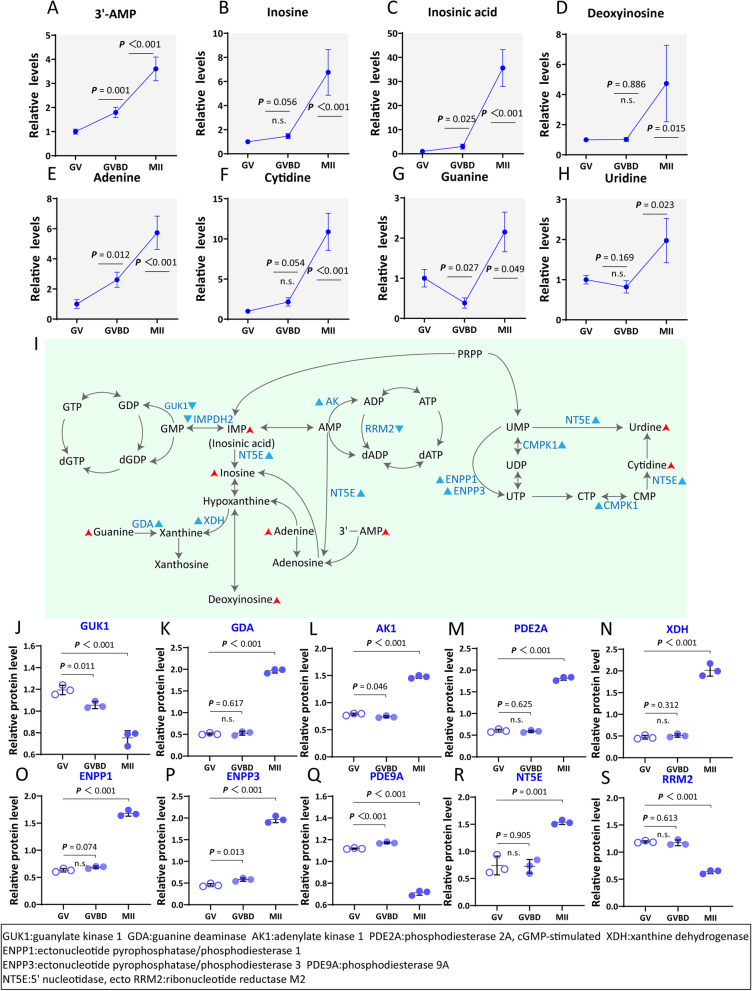
Progressive increase in nucleotide metabolism in cumulus cells during maturation. **A**-**H** Relative levels of metabolites related to nucleotide metabolism in cumulus cells. **I** Schematic diagram of nucleotide metabolism in cumulus cells during maturation. Metabolites increased in cumulus cells during meiotic resumption are indicated by bold red arrows. Differential metabolic enzymes changes are indicated by blue triangles. **J**-**S** Relative abundance of the representative enzymes involved in nucleotide metabolism. Error bars, SD. Student’s t test was used for statistical analysis in all panels, comparing to GV cumulus cells. n.s., not significant

Purine and pyrimidine nucleotides are the main form of energy utilization, involved in various physiological processes, such as cytokines (i.e., cAMP, cGMP), components of coenzymes (i.e., NAD + , FAD, CoA) and vehicles for the activation of intermediate metabolites(i.e., UDPG, CDP-DAG, SAM) [45]. During the COCs maturation, the levels of all detected nucleotide-related metabolites were gradually increased (i.e., 3’AMP, Inosine, Inosinic acid, Deoxyinosine, Adenine, Cytidine, Guanine and Uridine;) (Fig. 6A-H). Purine and pyrimidine metabolism originates from Ribulose 5-phosphate (R5P) and then progress into the synthesis of inosinic acid (IMP) (MII stage vs. GV stage: ~ 35 fold increase) and Inosine (MII stage vs. GV stage: ~ sevenfold increase). Similarly, the downstream metabolites of IMP were also enhanced during maturation (Fig. 6I). Meanwhile, integrated analysis of proteomics and metabolic pathways showed the accumulation of most enzymes associated with nucleotide metabolism in cumulus cells around mature oocytes (i.e., GDA, AK1, PDE2A, XDH, ENPP1, ENPP3 and NT5E*;*) (Fig. 6J-S). Collectively, the results clearly reveal the progressive increase in nucleotide metabolism in cumulus cells during oocyte maturation.

### Active amino acid metabolism in cumulus cells during maturation

**Fig. 7:**
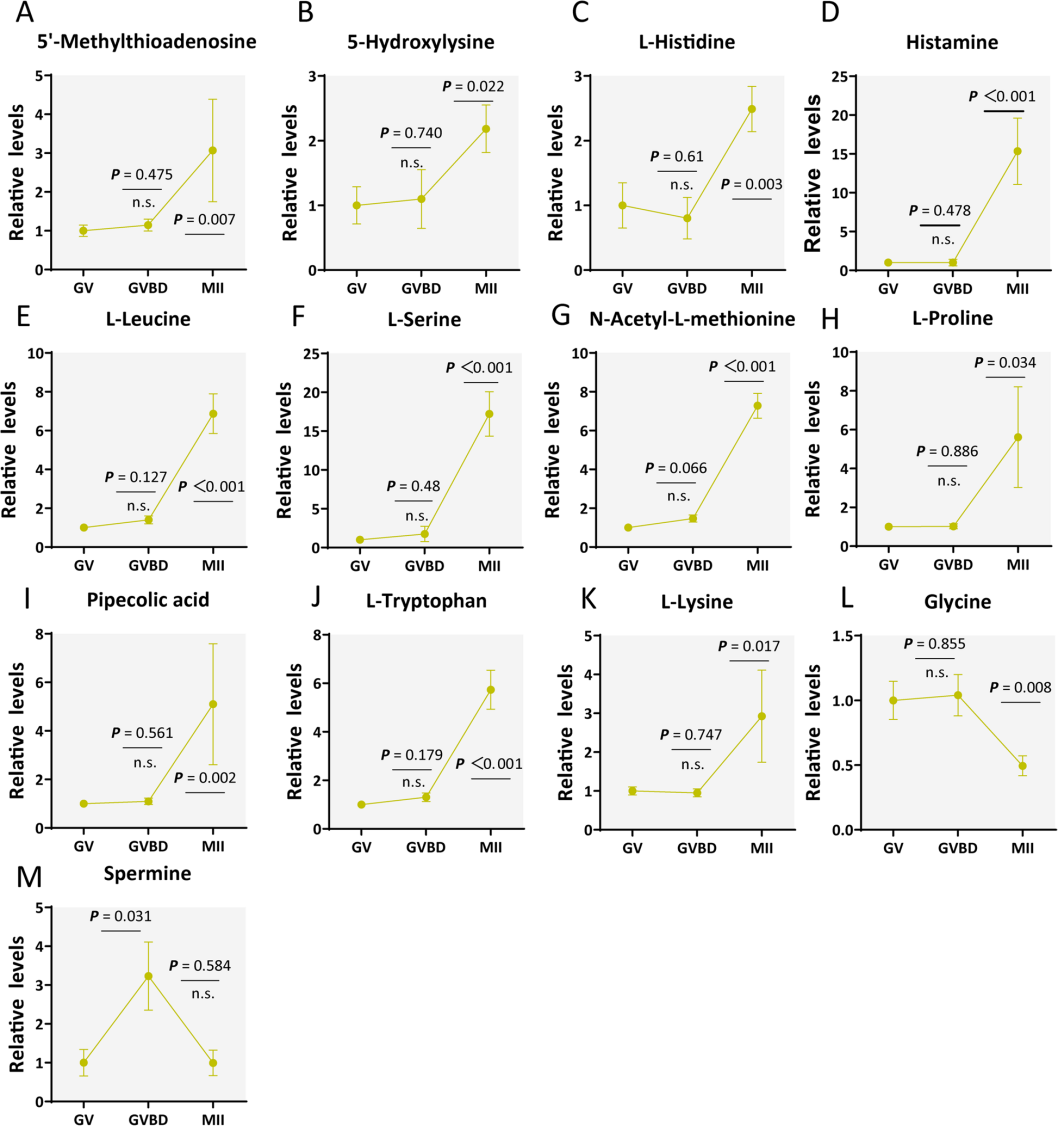
Amino acid metabolism in cumulus cells during maturation. **A**-**K** Relative levels of metabolites related to amino acid metabolism in cumulus cells at three time points. Error bars, SD. Student’s t test was used for statistical analysis in all panels, comparing to GV cumulus cells. n.s., not significant

As shown in Fig. 7A-M, 13 metabolites associated with amino acid metabolism were detected, and 11 metabolites (5'-Methylthioadenosine, 5-Hydroxylysine, L-Histidine, Histamine, L-Leucine, L-Serine, N-Acetyl-L-methionine, L-Proline, Pipecolic acid, L-Tryptophan, L-Lysine) were elevated in cumulus cells from meiotic resumption. ​Such an active metabolism strongly suggests that amino acid synthesis may be essential for healthy cumulus cell status, oocyte nutrient provision, and even the ovulation process. For example, histamine is a highly pleiotropic biogenic amine involved in key physiological processes including neurotransmission, immune response, cell differentiation, and inflammation [46]. In the cumulus cells surrounding MII oocytes, we observed that the levels of histamine and histidine are significantly increased (histamine: ~ 15-fold; histidine: ~ 2.5-fold) (Fig. 7C-D). It is conceivable that histamine may function as a potential regulator during ovulation. How amino acids in cumulus cells affect oocyte development remains to be thoroughly investigated.

### Metabolic cooperativity between cumulus cells and oocytes

**Fig. 8:**
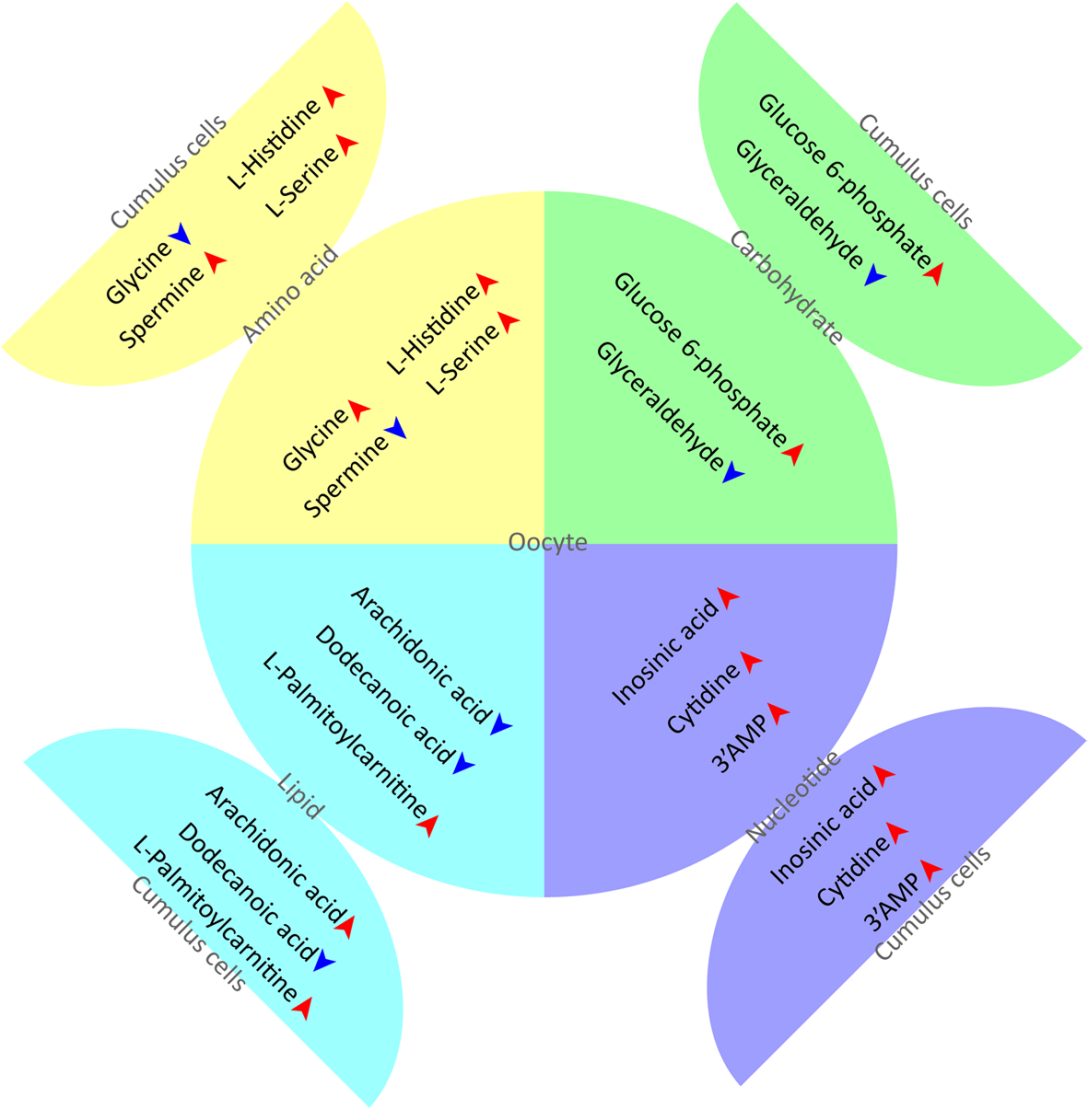
Metabolic cooperativity between cumulus cells and oocytes. (A) Metabolic tendency was compared between oocyte and cumulus cells. Severalrepresentative metabolites were presented in the diagram. Circles represent oocytes and semicircles represent cumulus cells, Metabolites increased are indicated by red arrows and decreased are indicated by blue arrows

Bi-directional communication between oocytes and the companion cumulus cells is essential for the development and functions of both compartments [47]. By comparing the metabolomic data between cumulus cells and oocytes, 21 metabolites were found in both groups [15]. Most of them are concentrated in nucleotide and amino acid metabolism (Fig. 8A). For instance, glucose-6-phosphate was found to be increased in both cumulus cells and oocytes during maturation. Considering that oocytes have low glycolytic activity, glucose-6-phosphate may be transported into oocyte from cumulus cells. Besides, in lipid metabolism, oocytes and cumulus cells displayed the similar metabolic tendency of dodecanoic acid and palmitoylcarnitine, and the opposite metabolic trend of arachidonic acid. The developmental significance of these metabolite changes would promote the understanding of metabolic coupling between oocyte and the surrounding somatic cells.

## Discussion

Here, we present an integrated analysis of metabolomics and proteomics by isolating mouse cumulus cells at three key stages, and illustrating the signatures of global metabolic patterns. ​Through multi-omics analysis, we identified a number of significantly altered metabolic pathways, including (i) enhanced hexosamine biosynthetic pathway, (ii) active steroid hormone generation, (iii) a dramatic synthesis of PGE2, [4] a progress increase in nucleotide metabolism. These dynamic changes in different pathways not only lay the foundation for comparative studies of cumulus cell development, but could also be used as metabolic signatures to predict the quality of COCs and improve in vitro culture systems for assisted reproduction.

Carbohydrate is an important material source of primary metabolism and secondary metabolism [48]. Glucose metabolized through various metabolic pathways is essential for meiotic maturation of mammalian oocytes, as it is used to generate purines and nucleic acids for DNA synthesis, NADPH for redox homeostasis, and hyaluronic acid for extracellular matrix formation during cumulus expansion [7]. Increased glycolysis in cumulus cells results in the high glucose-6-phosphate level. These results provide three potential explanations for the metabolic features in cumulus cells during oocyte maturation: (1) enhanced hexosamine biosynthetic pathway, (2) increased nucleotide sugar oxidation, (3) contribution to the synthesis of hyaluronic acid. In addition, changes in protein expression of GULO and SLC23A2 elaborate the source of ascorbic acid in cumulus cells. More than 40 million years ago, the inactivation of the GULO gene by a mutation left primates exclusively dependent on an exogenous dietary supply of ascorbic acid [49]. Recently, ascorbic acid has been shown to promote meiotic maturation and developmental competence in porcine oocytes [50, 51]. The construction of carbohydrate metabolism network in cumulus cells helps us to understand the metabolic pathways derived from glycolysis and provides a mechanistic framework to perceive how ascorbic acid affects the development of COCs.

Lipids are not only essential for the body's energy supply and storage, but they are also synthesized as steroid hormones to regulate metabolism [52]. In cumulus cells, we noted the increase of PGE2 and other steroid hormones during maturation. Rodents lacking the expression of key prostaglandin synthesis enzymes or treated with prostaglandin synthesis inhibitors experienced the reduced rates of cumulus expansion, follicle rupture, oocyte maturation and release [7]. Interestingly, the role of P4 and E2 in oocyte maturation has been controversial [53–55]. Steroid hormone metabolic disorders in obese or PCOS patients may be one of the main culprits in anovulation and impaired egg quality [56]. Here we have structured the spatial–temporal metabolic network of steroid hormones, providing potential targets for treatment and prevention of the related reproductive problems.

The oocyte and its surrounding cumulus cells are mutually dependent and regulate each other's metabolic functions in order to support the growth and maturation of COCs. Oocyte plays an active role in regulating the development and function of cumulus cells throughout the course of folliculogenesis [57]. For instance, there is substantial evidence that oocytes influence amino acid and lipid metabolism in cumulus cells [10, 11]. Clarification of the metabolic cooperativity between oocyte and cumulus cells will advance our understanding of the basic cellular and biochemical mechanisms controlling germ cell development, potentially identifying new avenues for augmenting oocyte quality and assessing oocyte developmental potential. One of the strengths of our study is the delineation of the metabolic pattern in in vivo-collected cumulus cells during maturation. In addition, the comparison of metabolite changes between oocyte and cumulus cells implicates the metabolic coordination. However, there are still some limitations in the present study. For example, we are not entirely sure whether the declining metabolites are required at low concentrations or consumed in large quantities [58]. Therefore, we inferred the metabolic flux based on the coordinated changes in both metabolite level and enzyme expression. Changes in metabolite levels occur through the alterations in metabolic enzyme accumulation (mRNA and protein level) as well as their activity, through allosteric regulation and protein modifications, such as phosphorylation and acetylation. In the present study, we only evaluated the enzyme abundance in cumulus cells, which perhaps partly explained the lack of correlation between enzyme expression and metabolite content in some metabolic pathways. Besides, this research is incapable of exploring the coupling or transportation of critical metabolites between oocyte and somatic cells, which deserves further assessment.

## Conclusion

Oogenesis is a complex physiological process that relies on well-balanced and timed metabolism. Multi-omics integration is key to a comprehensive understanding of complex biological processes. We have integrated data sets on metabolite and proteome dynamics not only to uncover the dynamic metabolic network in cumulus cells during oocyte maturation, but also to provide a new insight into the bi-directional communication network between cumulus cells and surrounding oocytes.

### Supplementary Information

**Additional file 1: Supplementary Table 1.** Related to Fig. 1. Metabolomic profiling in cumulus cells surrounding GV/GVBD/MII oocyte.

**Additional file 2: Supplementary Table 2.** Related to Fig. 2. Differentially Expressed Proteins in cumulus cells surrounding GV/GVBD/MII oocyte.

**Additional file 3: Figure S1.** Changes in the level of metabolic enzymes in cumulus cells during oocyte maturation. Related to Fig. 2E. Heat maps of relative levels of the indicated proteins in distinct metabolic pathways in cumulus cells during different stages of oocyte maturation.

**Additional file 4: Figure S2.** The same metabolites between Cumulus Cells and Oocytes. Related to Fig. 8A. The plot reflects the trend of the 21 metabolites in total.

## Data Availability

The mass spectrometry proteomics data have been deposited to the ProteomeXchange Consortium (http://proteomecentral.proteomexchange.org) via the iProX partner repository with the dataset identifier PXD040796.
