# Supplementary figures and images for: Multi-omics reveal the metabolic patterns in mouse cumulus cells during oocyte maturation
Source: J Ovarian Res. 2023 Aug 8;16:156. doi: 10.1186/s13048-023-01237-8 (PMC10408154; doi:10.1186/s13048-023-01237-8)

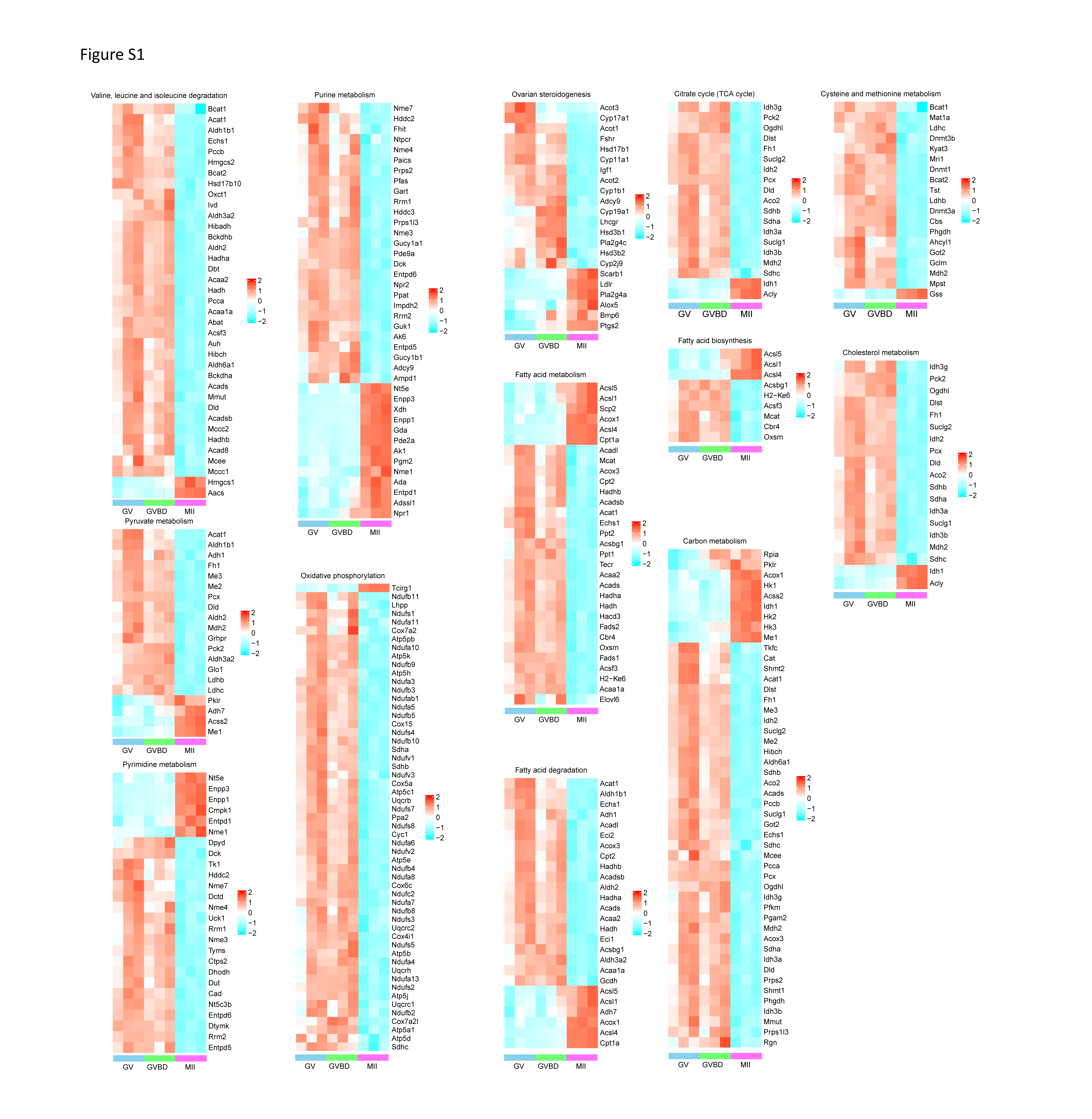

Supplement: Supplementary file 3 — Additional file 3: Figure S1. Changes in the level of metabolic enzymes in cumulus cells during oocyte maturation. Related to Fig. 2E. Heat maps of relative levels of the indicated proteins in distinct metabolic pathways in cumulus cells during different stages of oocyte maturation. [file 13048_2023_1237_MOESM3_ESM.tif]

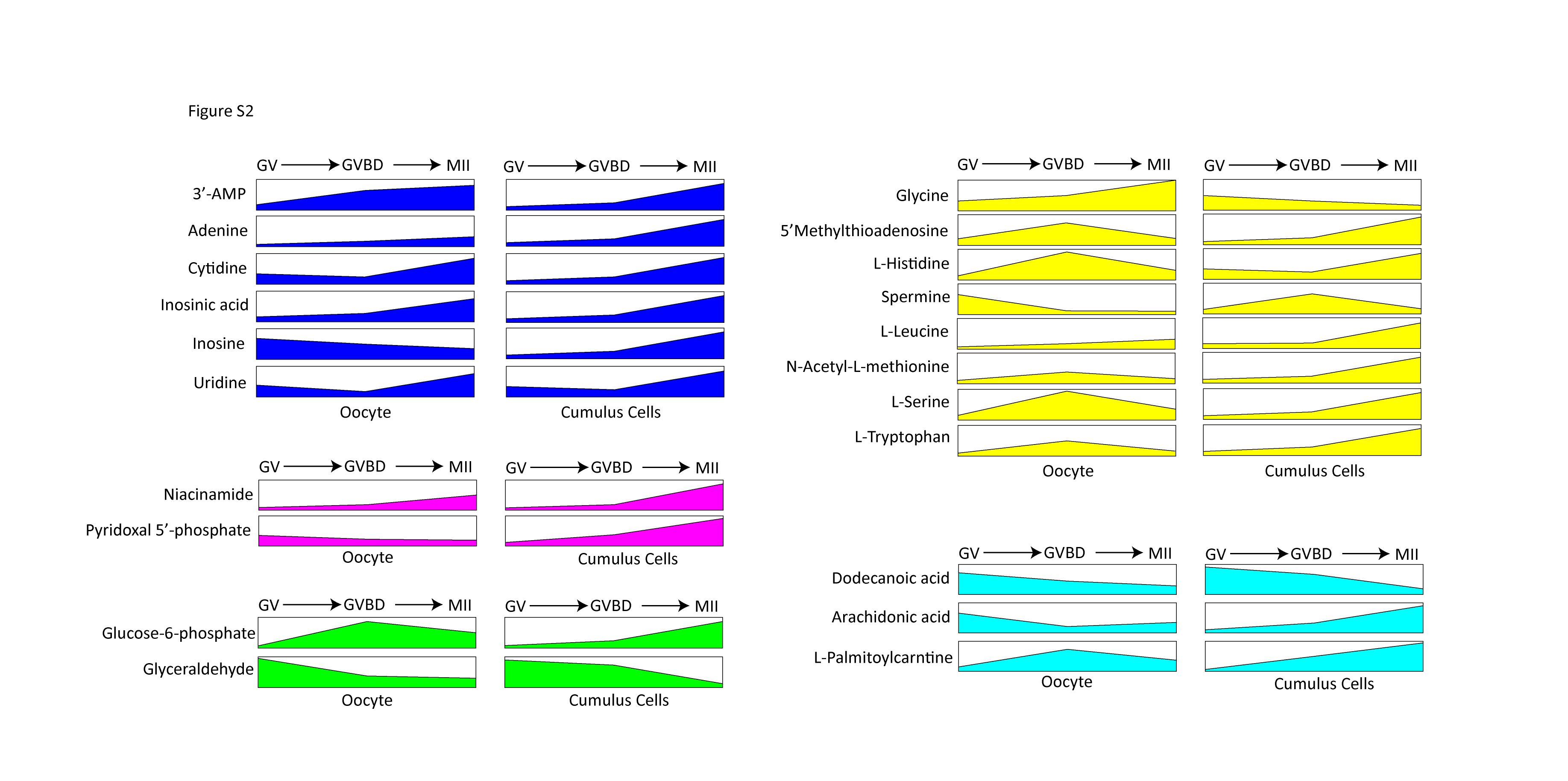

Supplement: Supplementary file 4 — Additional file 4: Figure S2. The same metabolites between Cumulus Cells and Oocytes. Related to Fig. 8A. The plot reflects the trend of the 21 metabolites in total. [file 13048_2023_1237_MOESM4_ESM.tif]
